# Magnetic resonance imaging in dental implant surgery: a systematic review

**DOI:** 10.1186/s40729-024-00532-3

**Published:** 2024-03-20

**Authors:** Adib Al-Haj Husain, Marina Zollinger, Bernd Stadlinger, Mutlu Özcan, Sebastian Winklhofer, Nadin Al-Haj Husain, Daphne Schönegg, Marco Piccirelli, Silvio Valdec

**Affiliations:** 1 Clinic of Cranio-Maxillofacial and Oral Surgery, Center of Dental Medicine, University of Zurich, Plattenstrasse 11, 8032 Zurich, Switzerland; 2 Department of Neuroradiology, Clinical Neuroscience Center, University Hospital Zurich, University of Zurich, Zurich, Switzerland; 3 Clinic of Chewing Function Disturbances and Dental Biomaterials, Center of Dental Medicine, University of Zurich, Zurich, Switzerland; 4 Department of Radiology, Hirslanden Zurich, Zurich, Switzerland; 5 Departement of Reconstructive Dentistry and Gerodontology, School of Dental Medicine, University of Bern, Bern, Switzerland; 6 Department of Oral and Cranio-Maxillofacial Surgery, University Hospital Basel, University of Basel, Basel, Switzerland

**Keywords:** Dental implants, Magnetic resonance imaging, Bone, Oral surgery, Oral radiology

## Abstract

**Purpose:**

To comprehensively assess the existing literature regarding the rapidly evolving in vivo application of magnetic resonance imaging (MRI) for potential applications, benefits, and challenges in dental implant surgery.

**Methods:**

Electronic and manual searches were conducted in PubMed MEDLINE, EMBASE, Biosis, and Cochrane databases by two reviewers following the PICOS search strategy. This involved using medical subject headings (MeSH) terms, keywords, and their combinations.

**Results:**

Sixteen studies were included in this systematic review. Of the 16, nine studies focused on preoperative planning and follow-up phases, four evaluated image-guided implant surgery, while three examined artifact reduction techniques. The current literature highlights several MRI protocols that have recently investigated and evaluated the in vivo feasibility and accuracy, focusing on its potential to provide surgically relevant quantitative and qualitative parameters in the assessment of osseointegration, peri-implant soft tissues, surrounding anatomical structures, reduction of artifacts caused by dental implants, and geometric accuracy relevant to implant placement. Black Bone and MSVAT-SPACE MRI, acquired within a short time, demonstrate improved hard and soft tissue resolution and offer high sensitivity in detecting pathological changes, making them a valuable alternative in targeted cases where CBCT is insufficient. Given the data heterogeneity, a meta-analysis was not possible.

**Conclusions:**

The results of this systematic review highlight the potential of dental MRI, within its indications and limitations, to provide perioperative surgically relevant parameters for accurate placement of dental implants.

**Graphical Abstract:**

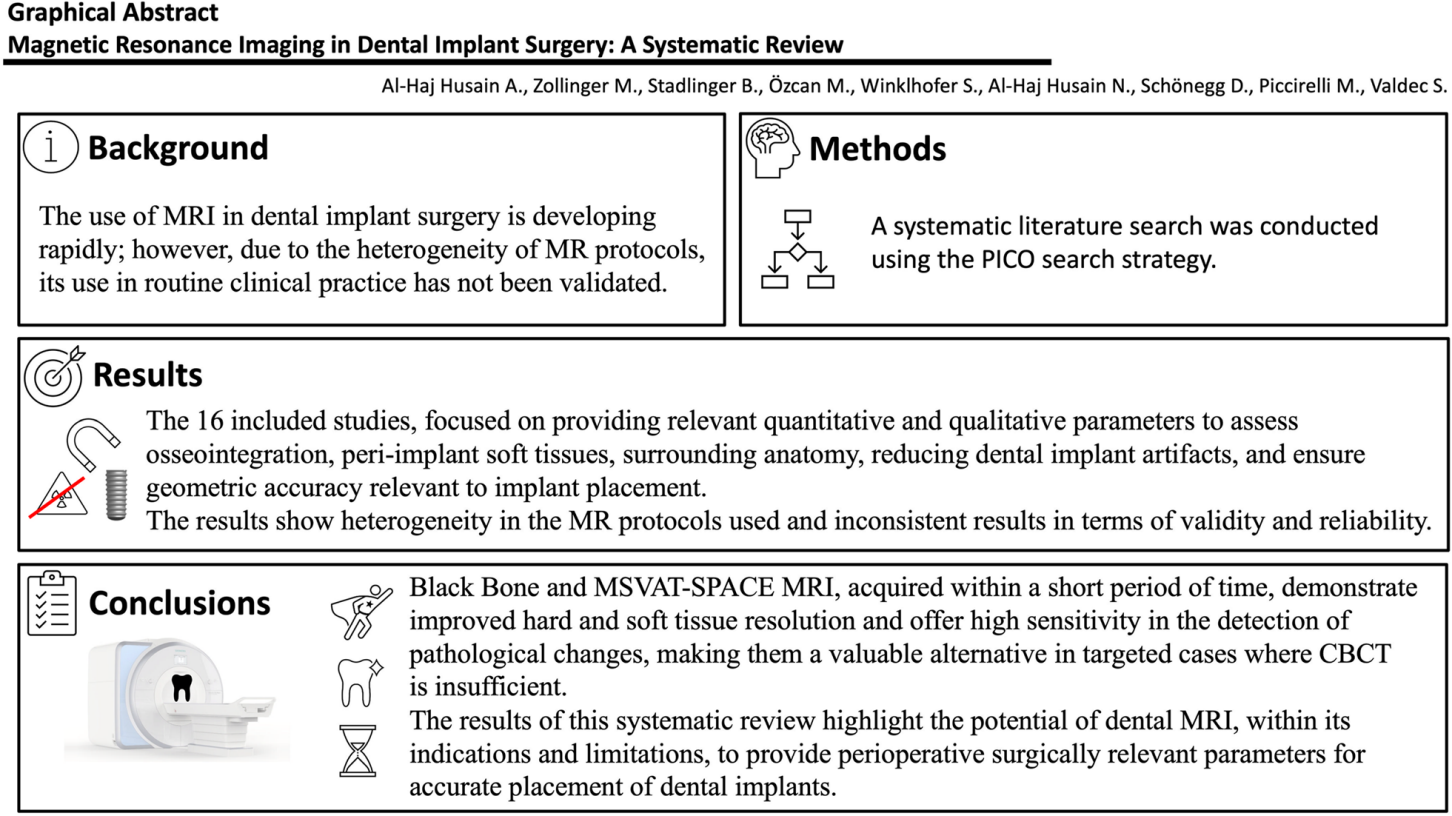

## Background

In recent years, three-dimensional cross-sectional imaging and navigation have made dental implant placement a popular and well-established treatment modality in modern dentistry. Taking into account medical, economic, psychological and social aspects, dental implants stand out as the optimal long-term solution for the replacement of one or more teeth with high survival rates [1]. Nonetheless, many local and systemic factors, such as patient-specific, implant-related, surgical technique, and environmental factors, are relevant and key to the long-term success of dental implant surgery [2].

In the pursuit of a multidisciplinary, personalized, and minimally invasive treatment approach, the comprehensive initial preoperative evaluation of the implant site commonly includes both clinical and radiographic assessments. Typically, a two-dimensional X-ray-based panoramic radiograph (PAN) is performed initially and is sufficient for many cases. In high-risk cases where it is challenging to accurately visualize important surgical details such as the quality and quantity of osseous structures, nearby vulnerable anatomical structures, or other concomitant pathologies, three-dimensional imaging such as cone-beam computed tomography (CBCT) is recommended [3]. Widely recognized as the gold standard for oral and maxillofacial solid tissue imaging, CBCT has further consolidated its integral role in dental implantology by becoming the imaging technique of choice for virtual surgical planning and subsequent CAD/CAM template fabrication for guided implant surgery [4]. Although experienced surgeons can achieve comparable results with freehand implant placement techniques, the incorporation of advanced biomedical imaging into the clinical workflow in combination with surgical planning has led to better outcomes in terms of positional accuracy while simultaneously improving the protection of vulnerable adjacent anatomical structures [5]. However, the use of CBCT in dental implantology comes with certain drawbacks, including radiation exposure, higher costs, potential overdiagnosis and overtreatment, and susceptibility to image artifacts, mainly in the presence of dense materials such as metals [4]. The increasing use of 3D imaging with higher radiation doses [4] may be associated with an elevated lifetime risk of radiation-induced tumors, particularly among genetically predisposed adolescents [6, 7]. Even though the age of the population undergoing dental implant surgery is generally higher, this stochastic radiation effect should always be taken into account, considering the continuous striving towards reducing or even eliminating radiation exposure in biomedical imaging according to the As Low As Reasonably Achievable (ALARA) principle and the upcoming suggested paradigm shift towards the As Low As Diagnostically Acceptable (ALADA) principle using CBCT [8].

Recently, magnetic resonance imaging (MRI), a non-invasive, non-ionizing radiation imaging modality, has undergone significant advancements in a wide range of technical improvements coupled with the development of novel MRI sequences, positioning it as a leading imaging modality for the head and neck region, especially for the visualization of soft tissues [9]. Given the increasing application of MRI in the dental field, it also represents a valuable tool for indication-specific implementation in perioperative dental implant diagnostics and treatment planning [10]. The shift towards radiation-free imaging techniques could potentially lead to more robust imaging regarding artifact susceptibility when dealing with metal or ceramic dental implants [11]. However, this transition is associated with challenges, as the pursuit of diagnostic accuracy must not be compromised. Challenges of MRI in dental implantology include high cost, limited accessibility, motion-induced distortions, accurate differentiation of complex anatomy with various small-sized nerves and blood vessels, and image distortion due to magnetic field inhomogeneities caused by dental restorations [12]. However, dental MRI protocols developed in recent years have made it possible to overcome limitations in imaging bone structures, one of the most essential parameters in dental implant surgery, by using, e.g., black bone MRI sequences [13] or ultrashort echo time (UTE) sequences [14]. Furthermore, integrating innovations such as intraoral [15] or mandibular coils [16] combined with specialized dental MRI protocols has led to novel high-resolution, high-contrast imaging of dentomaxillofacial structures within short acquisition times. The MRI signals generated can be digitized and combined so that variably mineralized hard and soft tissues can be simultaneously depicted, illustrating the potential emerging for improving perioperative diagnostics in dental implant surgery [17].

This systematic review aims to comprehensively assess the existing literature regarding the rapidly evolving in vivo application of MRI in dental implant surgery, explicitly emphasizing newly developed dental MRI protocols and technical innovations. By systematically evaluating the available evidence, this review aims to highlight the potential benefits, indication-specific limitations, and current evidence-based case-specific guidelines of MRI in the context of dental implantology. Additionally, this review aims to investigate the novel MRI protocols and techniques that have been developed to enhance their performance in assessing osseointegration, peri-implant soft tissues, and surrounding anatomical structures relevant to implant placement by addressing the following research question: Does the use of dental MRI and newly introduced MR protocols and techniques, considering their potential advantages and limitations, provide a comprehensive set of perioperative quantitative and qualitative diagnostic information for dental implant surgery in healthy subjects and patients?

## Materials and methods

### Search strategy

Following the guidelines of the preferred reporting items for systematic reviews and meta-analysis (PRISMA), this systematic review aimed to identify relevant studies by systematically conducting a comprehensive search using the following PICO (Population, Intervention, Comparison, Outcome) question: P-population: human studies in healthy volunteers or patients over 14 years of age undergoing perioperative dental MRI for dental implant surgery (endosteal implants, mini dental implants, orthodontic implants); I-intervention: magnetic resonance imaging; C-control: conventional radiological examination (e.g., PAN, CBCT, or computed tomography (CT)), if available; O-outcome: ensure the feasibility and diagnostic accuracy of perioperative radiographic evaluation in dental implant surgery, taking into account the acquisition of detailed images of dental and peri-implant structures with minimal artifacts (Table 1). This systematic review was not registered in the International Prospective Register of Systematic Reviews (PROSPERO) platform (no protocol number available).

**Table 1:** This systematic review aimed to identify relevant studies on the in vivo use of dental MRI in dental implantology by systematically conducting a comprehensive search using the following PICO (Population, Intervention, Comparison, Outcome) question

Focused Question (PICO)	Does the use of dental MRI and newly introduced MR protocols and techniques, considering their potential advantages and limitations, provide a comprehensive set of perioperative quantitative and qualitative diagnostic information for dental implant surgery in healthy subjects and patients?
*Search strategy*	
Population	Human studies (patients and/or healthy subjects), aged older than 12 years undergoing MRI prior to MTM surgery #1— “dental implants” OR “dental implant” OR “dental implantology” OR “titanium implant” OR “peri-implant” OR bone augmentation OR bone graft OR bone reconstruction OR sinus lift OR sinus lifting OR permanent dental restoration (inferior alveolar nerve [MeSH]) OR (lingual nerve [MeSH]) OR (mandibular nerve [MeSH]) OR (trigeminal nerve [MeSH]))
Intervention	Magnetic resonance imaging #2— ( (magnetic resonance imaging [MeSH]) OR (MRI) OR (nuclear magnetic resonance imaging [MeSH]) OR (NMR) OR (diffusion tensor imaging [MeSH]) OR (DTI) OR (ultra-short echo-time [MeSH]) OR (UTE) OR (maxillofacial imaging)) #3— ( (visualization) OR (neurography))
Comparison	Conventional preoperative radiological assessment #4— ( (computed tomography [MeSH]) OR (cone-beam computed tomography [MeSH]) #5— (panoramic radiography [MeSH])
Outcome	Feasibility and accuracy of perioperative radiological assessment in dental implant surgery #6— ( (accuracy) OR (feasibility) OR (signal-to-noise-ratio [MeSH]))
Search combination (s)	(#1) AND ( (#2 or #3 or #4 or #5) OR (#6))

### Information sources

A comprehensive data search of electronic databases for articles within the scope of this systematic review was conducted using Pubmed MEDLINE, EMBASE, BIOSIS, and Cochrane Library without imposing language restrictions. The search strategy was designed to target relevant articles published from 1993 until June 2023. The search syntax was divided into population, intervention, comparison, outcome, and study design, using primary keywords and Medical Subject Headings (MeSH) Terms and their combinations, while Boolean operators (AND, OR) were used to refine the search and identify relevant articles.

### Study selection and eligibility criteria

The studies included in this review were chosen based on the following criteria: (1) human studies, specifically clinical trials, involving healthy participants or patients undergoing MRI in mandibular or maxillary dental implant surgery, as part of randomized or nonrandomized controlled trials and cohort studies; (2) volunteers aged 14 years and older; (3) availability of the full text. Exclusion criteria were: (1) animal studies, cadaver studies, in-vitro studies utilizing designs employing non-biologic materials, commonly referred to as "phantoms'", along with systematic reviews, narrative reviews, and case reports; (2) patients with additional pathology at the surgical site; (3) if they focused on regions outside the maxilla or mandible; (4) if precise details of the use and timing of MRI imaging were not reported or were unclear. All data sourced from various databases were imported into EndNote 20 (Clarivate, Sydney, Australia), and subsequently, duplicate records were removed. Initially, the titles and abstracts were screened according to the inclusion and exclusion criteria, followed by a detailed full-text analysis. Two independent reviewers (A.A.H. and M.Z.) conducted the literature searches to minimize potential reviewer bias. Both reviewers thoroughly discussed and understood the specified inclusion and exclusion criteria. In addition, a training session was held between the reviewers to ensure the consistency in the interpretation and application of these criteria. If any discrepancies occurred during the screening process, they were resolved by discussion between the authors (A.A.H, M.Z., and S.V.).

### Data extraction and collection

For every study incorporated in this review, the subsequent data were recorded by two reviewers independently (A.A.H and M.Z.): general data (author details, year of publication, country, study design and objectives, sample size, and mean age and age range of participants), MRI-specific parameters (MR device utilized, MRI sequence (s), field strength, type of MR coil, and acquisition time), and outcome measures (feasibility and diagnostic accuracy of perioperative radiographic evaluation, taking into account the acquisition of detailed images of dental and peri-implant structures with minimal artifacts).

### Risk-of-bias assessment and quality assessment of studies

The assessment of the risk of bias in the methodology of the studies incorporated in this systematic review was assessed based on a modified short version of the Strengthening the Reporting of Observational Studies in Epidemiology (STROBE), as outlined by Edwards et al. [18]. The evaluation compromised 18 criteria from the STROBE statement. Studies with a total score of 15 or more out of 18 were considered to have a low risk of bias, while those scoring between 11 and 14 were considered to have a medium risk of bias. Studies with a score of 10 or less were categorized as having a high risk of bias.

The Quality Assessment of Diagnostic Accuracy Studies (QUADAS-2) [19] tool, which compromises four domains (patient selection, index test, reference standard, and flow and timing), was used to assess and ensure a transparent evaluation of bias, methodological soundness, diagnostic quality and applicability of primary diagnostic accuracy studies.

## Results

### Study selection

**Fig. 1:**
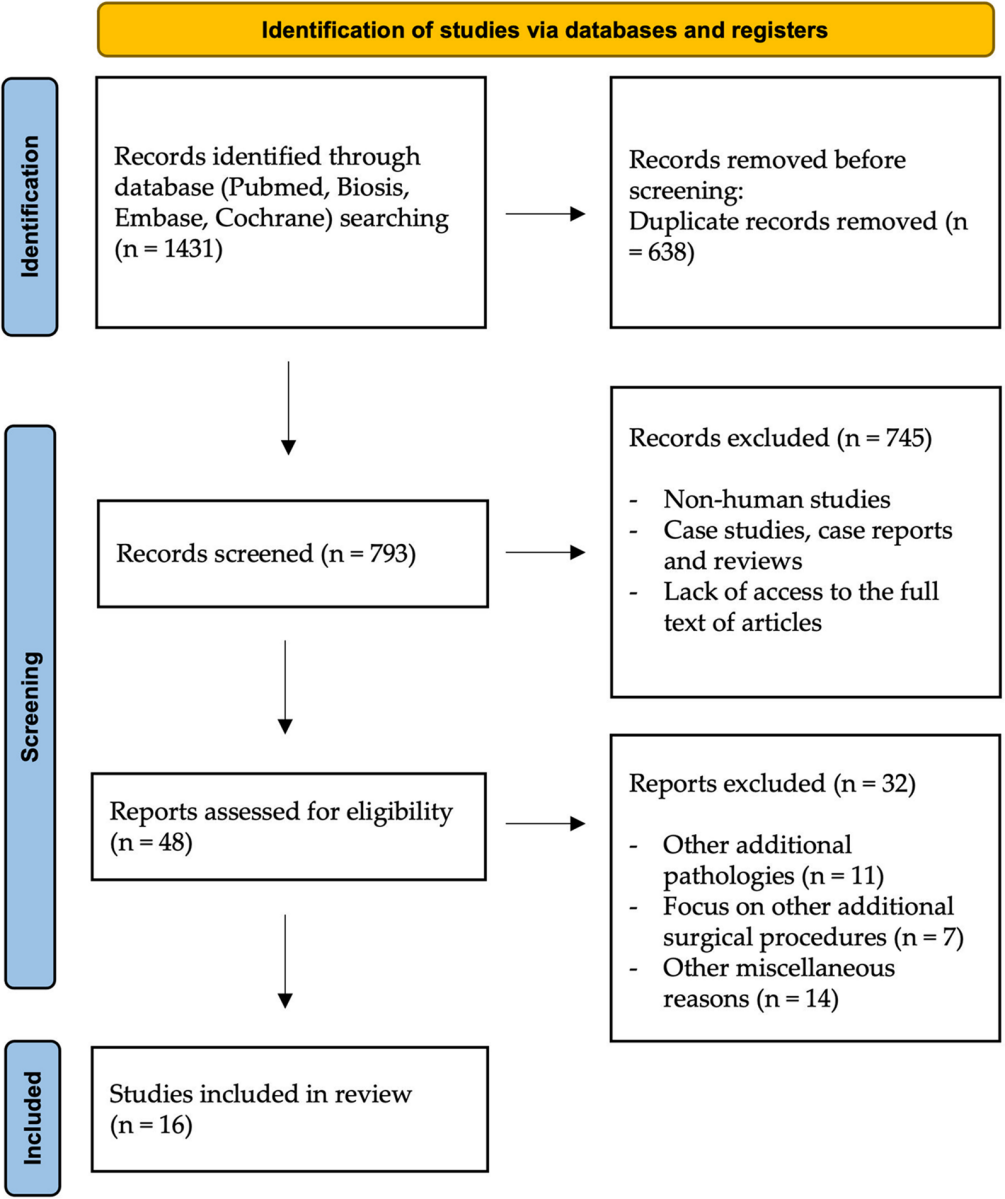
Preferred reporting items for systematic reviews and meta-analysis (PRISMA) flow diagram showing the article selection in this review

According to the aim of this comprehensive review, the systematic literature search initially identified 1431 studies of potential relevance. After removing all duplicates, 793 articles remained. In the first step of study selection, the titles and abstracts were screened, which resulted in the exclusion of 745 articles. This left 48 articles for a thorough full-text evaluation in the subsequent analysis step. Of these, 32 articles were excluded because they did not meet the inclusion criteria. Finally, 16 articles dealing with the in vivo use of MRI in dental implant surgery fell within the scope of this systematic review and were evaluated (Fig. 1). The final selection of the studies included in this analysis was thoroughly reviewed and approved by the rest of the remaining authors.

### Study characteristics

From an initial pool of 1431 articles of potential relevance, a total of 16 articles successfully met the inclusion criteria after a comprehensive analysis of their full texts. These articles, authored by Gray et al., 1998 [21]; Hassfeld et al., 2001 [22]; Imamura et al., 2004 [24]; Senel et al., 2006 [34]; Pompa et al., 2010 [23]; Burger et al., 2015 [55]; Probst et al., 2017 [52]; Laurino et al., 2020 [32]; Arabi et al., 2020 [54]; Probst et al., 2020 [13]; Hilgenfeld et al., 2020 [42]; Flügge et al., 2021 [33]; Schwindling et al., 2021 [44]; Grandoch et al., 2021 [31]; Schwindling et al., 2021 [56]; Al-Haj Husain et al., 2023 [16], were considered directly relevant to the scope of this review. In these 16 selected studies (comparative study 75%; other 25%), published between July 1998 and June 2023, a total of 269 patients underwent perioperative MRI. These scans were performed using MRI field strengths of 0.2, 1.5, or 3 Tesla and different MRI sequences. Patients ranged in age from 14 to 85 years, and scan acquisition times varied from approximately 3 min to 21 min.

Nine of the included studies addressed bone-specific features relevant to osseointegration in the preoperative planning and follow-up phases, such as bone level assessment in general or in specific anatomical conditions, e.g., maxillary and mandibular alveolar atrophy at the implant site, perioperative assessment in sinus lift surgery or onlay bone grafting, or assessment of buccal bone thickness in planning of immediate implant placement in the anterior region. Four studies evaluated fully or partially image-guided implant surgery, three assessed artifact reduction, one evaluated artifact reduction caused by dental implants in specific dental MR protocols, and two evaluated artifact reduction in hybrid PET/MRI imaging, including deep learning-based approaches. The characteristics of the studies included in this systematic review are presented in Table 2.

**Table 2:** Characteristics of included studies in this systematic review

Study number	Author, Year, Country	Study	Sample size	Mean age; (range)	Study objectives	MRISequences	Number and type of implants	Field strengths	Type of MRI coil	Acquisition time	MR device	Outcome parameters (feasibility/accuracy)
1	Gray et al., 1998, Scotland, United Kingdom	Low-field magnetic resonance imaging for implant dentistry	11 Patients	N/A	Evaluation of the available bone level for dental implant placement	T1-weightedfast spin echo (SE) technique	19 implants (13 maxillary and six mandibular);titanium	0.2 T	Radiofrequency head coil	5:16 min:s	Open Viva, Siemens AG, Erlangen, Germany	Accurate preoperative visualization of the implant site, distinguishing between cortical and cancellous bone and associated anatomical structures, with minimal artifacts
2	Hassfeld et al., 2001, Germany	Magnetic resonance tomography for planning dental implantation	15 patients	40 years; (14–85 years)	Assessment of MRI for pre-implant imaging inpatients with severe alveolar atrophy	T1-weighted, fat-suppressed sequences and conventional T1-weighted sequences	N/A	1.5 T	N/A	14:09 min:s	Edge High-Field Magnetic Resonance Imaging System, Picker, Cleveland, USA	Detailed depiction of anatomically relevant structures, such as the mandibular canal and maxillary sinus. Artifacts caused by metallic materials reduced the image quality
3	Imamura et al., 2004, Japan	A Comparative Study of ComputedTomography and Magnetic Resonance Imaging for the Detection of Mandibular Canals and Cross-Sectional Areas in Diagnosis prior to Dental Implant Treatment	11 Patients	59 years; (35–75 years)	Evaluation and comparison of the detectability of the anatomical morphology of mandibular molar implant sites using CT and MRI prior to dental implant treatment	T1-weightedMRI	19 implants;N/A	1.5 T	N/A	N/A	MAGNEX 150™, Shimadzu Corporation, Kyoto, Japan	MRI identified the canal in all cases, while CT failed to do so in half of the cases, with high inter- and intra-reader reliability
4	Senel et al., 2006, Turkey	Assessment of the sinus lift operation by magnetic resonance imaging	8 patients	N/A;(38–55 years)	Evaluation of theedentulous maxillary regions one week before and three months after sinus lift surgery	T2-weighted fast spin echo (FSE),T1-weighted spin echo (SE)	13 implants;N/A	1.5 T	N/A	10 min	N/A	High-resolution visualization of the surgical site for preoperative planning and postoperative vertical bone height augmentation
5	Pompa et al., 2010, Italy	A comparative study of Magnetic Resonance (MR) and Computed Tomography (CT) in the pre-implant evaluation	30 patients	N/A	Evaluation and comparison of the bone level for pre-implant evaluationby CT and MRI	Fast-gradient-echo sequence; Proton density (PD)-weighted, T2-weighted and Tl-weighted spin-echo sequences	N/A; Prior Implants (amalgam,ferrous)	1.5 T	Head and neck coil	9 min	N/A	Accurate visualization and reliable bone measurements in the surgical site for preoperative dental implants, with no significant differences found between both imaging modalities
6	Burger et al., 2015, Switzerland	Hybrid PET/MR Imaging: An Algorithm to Reduce Metal Artifacts from Dental Implants in Dixon-Based Attenuation Map Generation Using a Multiacquisition Variable-Resonance Image Combination Sequence	8 patients	N/A	Development of an algorithm that adapts Dixon MR-based imaging to minimize metal artifacts from dental implants in hybrid PET/MR imaging	3-Dimensional dual gradient-echo sequence (Dixon) used for MR imaging–based PET attenuation correction and a high-resolution multiacquisition with variable resonance image combination (MAVRIC) sequence	N/A; titanium	3 T	N/A	6:38 min:s	Discovery MR750, imaging scanner, General Electric Healthcare, Milwaukee, Wisconsin, USA	The proposed algorithm was robust in all patients and allowed a significant 70% reduction in artifact size, allowing MR image-based attenuation correction in critical areas
7	Probst et al., 2017, Germany	Magnetic resonance imaging of the inferior alveolar nerve with special regard to metal artifact reduction	7 patients	N/A	To identify the potential and limitations of postoperative MRI of the inferior alveolar nerve (IAN) in dental implant surgery, especially regarding metal artefacts	Three-dimensional (3D) turbo spin echo (TSE) and gradient echo (GRE) sequences, T1-weighted volumetric interpolated breath-hold examination (VIBE) with fat suppression, and Constructive Interference in Steady State (CISS) with a high T2 contrast, WARP sequences	N/A;metallic materials	1.5 or 3 T	12-channel head coil with an additional surface coil	20:58 min:s	MAGNETOM Verio, Siemens Healthcare, Erlangen, GermanyMAGNETOM Avanto, Siemens Healthcare, Erlangen, Germany	Subjects with postoperative neurosensory IAN impairment showed a significant reduction in metallic artifacts. The use of view angle technique (VAT) and slice-encoding metal artifact correction (SEMAC) techniques further improved image quality, but was associated with a blurring effect
8	Laurino et al., 2020, Brazil	Correlation between magnetic resonance imaging and cone-beam computed tomography for maxillary sinus graft assessment	15patients	59 years; (N/A)	Quantitative and qualitative assessment of postoperative bone dimensions after unilateral sinus lift surgery using CBCT and MRI	T1-weighted spin-echo sequence, T2-weighted spin- echo sequence	N/A	1.5 T	Head coil	N/A	MAGNETOM Aera; Siemens Healthcare, Erlangen, Germany	The presence of bone tissue in the grafted area was observed, with significant correlations between MRI and CBCT for sinus graft height, buccolingual width, and anteroposterior depth
9	Arabi et al., 2020, Switzerland	Truncation compensation and metallic dental implant artefact reduction in PET/MRI attenuation correction using deep learning-based object completion	25 patients	65 years; (50–77 years)	Application of a deep learning-based assessment to predict the missing information in MR images compromised by metallic artifacts due to dental implants, with the aim of reducing quantification errors in PET/MRI	Dixon 3D volumetric interpolated T1-weighted sequence	N/A; metallic materials	3 T	N/A	N/A	Ingenuity TF PET/MRI system, Philips Healthcare, Cleveland, Ohio, USA	The results show promising performance of the proposed approach and reduction of artifacts in completing MR images compromised by metal artifacts and/or body truncation in PET/MR imaging
10	Probst et al., 2020, Germany	Magnetic resonance imaging based computer-guided dental implant surgery—A clinical pilot study	12 patients	49 years; (N/A)	Evaluation of the feasibility of computer-assisted template-guided 3D dental implant planning is feasible using MRI	3D T1-weighted bone sequence, 3D T2-weighted short tau inversion recovery (STIR)	12 implants; N/A	3 T	16-channel Head and Neck Spine array	9:11 min:s	MR Ingenia Elition, Philips Healthcare, Best, the Netherlands	MRI-based guided dental implant surgery was feasible in 75% of the cases, with the resulting deviations between the virtually planned and the actual implant position being clinically acceptable
11	Hilgenfeld et al., 2020, Germany	Use of dental MRI for radiation-free guided dental implant planning: a prospective, in vivo study of accuracy and reliability	30 patients	57 years; (N/A)	MRI datasets were used for implant planning and surgical guide fabrication in patients undergoing dental implant surgery. In addition, CBCT datasets were used to co-register and evaluate angular discrepancies between the planned and surgically guided positions of the implants	Multi-slab acquisition with view-angle tilting gradient was used, based on a sampling perfection with application-optimized contrasts using different flip-angle evolution (MSVAT-SPACE) prototype sequence	45 implants; N/A	3 T	15-channel dental coil	7:45 min:s	MAGNETOM Tim Trio, Siemens Healthcare, Erlangen, Germany	Inter-rater and inter-modality agreement was excellent for MRI-based treatment planning. CBCT-based adjustments to MRI plans were required for implant position at 30% and implant axis at 7%, with almost all guides being suitable for clinical use
12	Flügge et al., 2021, Germany	MRI for the display of autologous onlay bone grafts during early healing—an experimental study	10 patients	52.5 years; (26–64 years)	Assessment of graft volume of autologous onlay bone grafts during early healing in patients with alveolar bone atrophy	2D Turbospinecho (TSE) sequences with view angle tilting (VAT) technique	N/A	3 T	Body transmit coil, a 4 cm receive loop coil (LC), and an intraoral inductively coupled coil (ICC)	2:38–5:03 min:s	MAGNETOM Prisma, Siemens Healthineers, Erlangen, Germany	MRI is capable of accurately imaging autologous onlay bone grafts longitudinally, but in some cases image artifacts have caused volumetric measurement deviations
13	Schwindling et al., 2021, Germany	Three-dimensional accuracy of partially guided implant surgery based on dental magnetic resonance imaging	34 patients	57 years; (29–75 years)	Quantifying the three-dimensional accuracy of partially guided implant surgery using backward planning, based on dental magnetic resonance imaging	Multi-slab acquisition with view-angle tilting gradient was used, based on a sampling perfection with application-optimized contrasts using different flip-angle evolution (MSVAT-SPACE) prototype sequence	41 implants; N/A	3 T	15-channel dental coil	10 min	MAGNETOM Tim Trio, Siemens Healthcare, Erlangen, Germany	The 3D accuracy of MRI-guided partially guided implant surgery was lower for entry point, apex and axis than for CBCT-guided. Nevertheless, the values are promising for radiation-free backward planning
14	Grandoch et al., 2021, Germany	1.5 T MRI with a Dedicated Dental Signal-Amplification Coil as Noninvasive, Radiation-Free Alternative to CBCT in Presurgical Implant Planning Procedures	16 patients	N/A; (19–78 years)	Evaluation of dental MRI as a radiation-free alternative for dental implant planning using a dedicated dental signal amplification coil and to compare it with CBCT	3D high-resolution T1-weighted turbo- spin echo sequence (3D HR T1w TSE), 3D high resolution T1- weighted fast field echo sequence (3D HR T1w FFE)	22 implants; N/A	1.5 T	Orbital 4-channel coil	8:52 min:s	Philips Achieva, Philips Healthcare, Best, the Netherlands	Dental Implant planning was technically feasible by all MRI protocols, whereby 3D HR T1w TSE was superior and showed no significant differences compared to CBCT
15	Schwindling et al., 2021, Gerrmany	Geometric Reproducibility of Three-Dimensional Oral Implant Planning Based on Magnetic Resonance Imaging and Cone-Beam Computed Tomography	27 patients	N/A	Evaluation of geometric reproducibility of 3D implant planning based on MRI and CBCT using a backward planning approach and assessment of inter- and intra-rater reliability	Multi-slab acquisition with view-angle tilting gradient was used, based on a sampling perfection with application-optimized contrasts using different flip-angle evolution (MSVAT-SPACE) prototype sequence	41 implants; N/A	3 T	15-channel dental coil	10 min	MAGNETOM Tim Trio, Siemens Healthineers; Erlangen, Germany	CBCT-based implant planning was more reproducible than MRI and inter- and intra-rater reliability was higher with CBCT than with MRI
16	Al-Haj Husain et al., 2023, Switzer-land	Buccal bone thickness assessment for immediate anterior dental implant planning: A pilot study comparing cone-beam computed tomography and 3D double-echo steady-state MRI	10 patients	32 years; (19–59 years)	CBCT vs. MRI evaluation of buccal bone thickness for anterior implant planning	3-dimensional double-echo steady-state (DESS) MRI	N/A	3 T	64 channel head-and-neck coil	12:24 min:s	Skyra (release VE11c), Siemens Healthineers, Erlangen, Germany	Image quality showed little to no artifacts and allowed confident diagnostic interpretation, with no significant differences in buccal bone thickness assessment between both imaging modalities

### Risk-of-bias assessment and quality assessment of studies

**Fig. 2:**
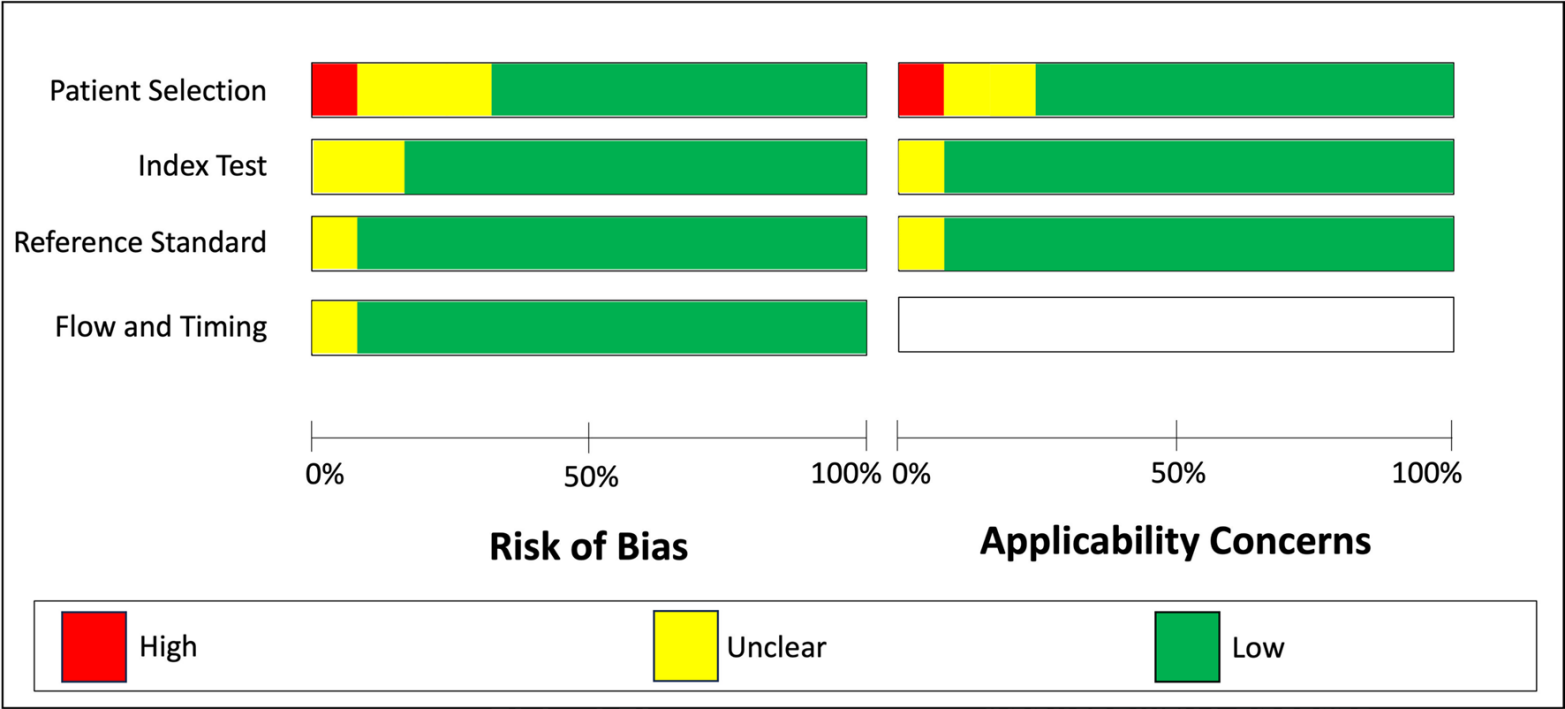
Study risk of bias and applicability concerns assessment using the Quadas-2 (Quality Assessment of Diagnostic Accuracy Studies 2) tool

The risk of bias was assessed as low in 14, medium in one study, and high in one study. An overview of the percentage responses for each topic is displayed in Table 3. The assessment of diagnostic quality and applicability according to the QUADAS-2 rules is visualized in Fig. 2.

**Table 3:** Quality assessment of the risk of bias in the methodology of the studies incorporated in this systematic review was assessed based on a modified short version of the Strengthening the Reporting of Observational Studies in Epidemiology (STROBE) (+ = yes, − = no)

Checklist	Gray et al., 1998 (21)	Hassfeld et al., 2001 (22)	Imamura et al., 2004 [24]	Senel et al., 2006, [34]	Pompa et al., 2010 [23]	Burger et al., 2015 [55]	Probst et al., 2017 [52]	Laurino et al., 2020 [32]	Arabi et al., 2020 [54]	Probst et al., 2020 [13]	Hilgenfeld et al., 2020 [42]	Flügge et al., 2021 [33]	Schwindling et al., 2021, [56]	Grandoch et al., 2021 [31]	Schwindling et al., 2021, [44]	Al-Haj Husain et al., 2023 [17]
*Objectives:* clearly formatted	+	+	+	+	+	+	+	+	+	+	+	+	+	+	+	+
*Study design:* described in detail	+	+	+	+	+	+	+	+	+	+	+	+	+	+	+	+
*Settings:* described in terms of location; and relevant dates	−; −	+ ; +	+ ; +	+ ; +	+ ; +	+ ; +	+ ; +	+ ; +	+ ; +	+ ; +	+ ; +	+ ; +	+ ; +	+ ; +	+ ; +	+ ; +
*Participants:* eligibility criteria; and methods of selection described	−; −	−; −	+ ; +	+ ; +	+ ; +	+ ; +	+ ; +	+ ; +	+ ; +	+ ; +	+ ; +	+ ; +	+ ; +	+ ; +	+ ; +	+ ; +
*Bias:* any efforts to address potential sources of bias described	+	+	+	+	+	+	+	+	+	+	+	+	+	+	+	+
*Sample size:* explanation of derivation; adequate	−; +	−; +	−; +	−; +	−; +	−; +	−; −	+ ; +	−; +	−; +	−; +	−; +	−; +	−; +	−; +	−; +
*Statistical Methods:* described; appropriate for data	−; +	+ ; +	+ ; +	−; +	+ ; +	+ ; +	+ ; +	+ ; +	+ ; +	+ ; +	+ ; +	+ ; +	+ ; +	+ ; +	+ ; +	+ ; +
*Participants:* described	−	+	+	+	−	−	+	+	+	+	+	+	+	+	+	+
*Outcome data:* number of outcome events reported	+	+	+	+	+	+	+	+	+	+	+	+	+	+	+	+
*Other analysis:* any other analyses conducted reported	−	+	−	−	−	−	+	−	−	−	+	−	−	+	+	−
*Limitations:* limitations of the study; and any potential bias discussed	−; +	+ ; +	−; +	−; +	+ ; +	+ ; +	+ ; +	+ ; +	+ ; +	+ ; +	+ ; +	+ ; +	+ ; +	+ ; +	+ ; +	+ ; +
*Interpretation:* overall interpretation of results provided	+	+	+	+	+	+	+	+	+	+	+	+	+	+	+	+
*External validity:* generalizability of the results discussed	+	+	+	+	+	+	+	+	+	+	+	+	+	+	+	+
Total out of 18 (percentage)	9 (50%)	15 (83%)	15 (83%)	14 (78%)	15 (83%)	15 (83%)	17 (94%)	17 (94%)	16 (89%)	16 (89%)	17 (94%)	16 (89%)	16 (89%)	17 (94%)	17 (94%)	16 (89%)

## Discussion

Numerous articles in the literature have investigated and evaluated the feasibility and accuracy of MRI in dental implantology, focusing on its potential to provide surgically relevant quantitative and qualitative parameters. Subsequently, the analysis in this review has focused on the impact of MR protocols and device-specific technical features in perioperative imaging, aiming to assess their indications and limitations and provide recommendations for the most appropriate decision-making. The results of this review highlight the significant contribution of dental MRI to the comprehensive assessment of surgically relevant clinical parameters of the implant site, including details of the osseous tissue structure, bone dimensions (height and width), proximity to the mandibular canal, respectively nerves and foramina, and delineation of the osseous boundaries of the maxillary sinus. This is particularly important in cases where conventional imaging techniques such as CBCT may be inadequate due to limitations in visualizing soft tissue structures.

For more than three decades, MRI has been used to image the pathoanatomy of the dentomaxillofacial region, allowing simultaneous radiation-free visualization of soft and hard tissues [20]. Several previously conducted clinical studies have demonstrated that MRI has the potential to be used for dental implant planning, even though the aspect of template-guided implant positioning was not initially considered [12, 21–23]. Nonetheless, the long scan times of up to 30 min and suboptimal image quality with insufficient resolution at slice thicknesses of up to 4 mm have, until recently, proved unsuitable for daily clinical routine. However, the data obtained support that MRI using conventional MRI sequences (T1-weighted gradient‐echo (GE) and fast spin echo (SE) sequences) has been shown to provide similar, non-inferior information to CBCT or CT in the preoperative planning of dental implants [23, 24]. It should be noted that the conventional MRI protocols used in these studies allowed the acquisition of signals from nonmineralized soft tissues but could not directly visualize mineralized crystalline tissues such as teeth or bone, presenting them as dark voids or regions devoid of signal. In clinical practice, X-ray-based three-dimensional sectional imaging modalities, particularly CBCT, have established themselves as the gold standard for patient-specific preoperative virtual surgical planning, and the subsequent fabrication of CAD/CAM-generated drilling templates for guided implant surgery [4, 25]. The use of planning software enables the virtual positioning of implants, taking into account prosthetic and anatomical considerations and the available bone level so that the virtually planned implant position can be transferred to the surgical site with appropriate clinical accuracy [25]. However, with the increasing use of CBCT in medical imaging, potential concerns have been raised due to cumulative radiation exposure, resulting in an increased susceptibility to thyroid cancer and meningiomas [26, 27]. Ongoing research is still being conducted to understand further the exact relationship between radiation exposure and the implementation of X-ray-based scans in routine clinical dental procedures.

Recent developments advancing the field of dental imaging towards innovative MRI protocols optimized specifically for dental applications, along with the integration of dedicated novel coils, have enabled the acquisition of high isotropic 3D resolution, with significant improvement in resolution, signal-to-noise ratio, contrast-to-noise ratio, as well as significant reduction in acquisition times and effective suppression of artifacts [15, 28, 29]. As a result, MRI is emerging as a promising and reliable alternative to CBCT for dental implant surgery, both for accurate diagnosis and perioperative treatment planning, when indicated. Previous studies have shown that CT, CBCT, and MRI have excellent intermodal agreement for dimensional and positional measurements, including parameters such as bone height and width and bone dimensional volume [23, 29–31].

Especially in the perioperative assessment of sinus lift surgery [32] or onlay bone grafting [33], excellent results seem to be obtained with a significant correlation between MRI and CBCT. Sinus floor elevation procedures showed the best intermodality agreement for sinus graft height, buccolingual width, and anteroposterior depth [32, 34], whereas onlay bone grafts allowed accurate longitudinal visualization, with image artifacts in some cases causing volumetric measurement deviations [33]. Thus, it can be concluded that MRI allows accurate assessment of the outcome of sinus floor elevation with high image quality and little or no artifacts. Mineralized dental tissue is challenging to depict using conventional MRI sequences due to its low proton density and biological composition, which limit the molecular motion of hydrogen nuclei within water molecules, coupled with rapid signal decay after radiofrequency excitation [35, 36]. For effective visualization of teeth and critical bony structures, such as the osseous boundaries of the maxillary sinus and mandibular canal, along with the inferior alveolar nerve, specialized sequences are required. These sequences should be capable of acquiring rapidly decaying signals, a feat achieved by implementing ultra-short echo time (UTE) and zero echo time (ZTE) techniques. These techniques offer the advantage of generating CT-like contrast while simultaneously enabling the visualizing soft tissue signals [37, 38]. Thereby, ultrashort hard pulse excitations and three-dimensional center-out radial sampling are utilized, resulting in high-quality imaging. Due to its ultra-short echo time, the UTE protocol is also particularly well suited for reducing metal or field inhomogeneity artifacts, highlighting its enormous potential in dental implant imaging [14]. Regarding the visualization of neural tissues, especially the continuous depiction of the trigeminal nerve and its peripheral branches, such as the inferior alveolar or lingual nerve, the combination of black bone MRI sequences, such as 3D-double echo steady state (DESS) sequences with novel mandibular coils provides excellent perioperative imaging of the surgical site [9], [39]. For high-risk procedures near vulnerable soft tissue anatomy, such as vessels, nerves, gingiva, and adjacent periodontal ligaments, DESS MRI could enhance the procedure's safety and improve patient outcomes. However, both CBCT and MRI can have limitations in terms of accurate visualization of the occlusal surfaces, which is required for precise tooth-guided implant positioning. Both in vitro and in vivo studies are currently demonstrating the feasibility and accuracy of the MRI-based approach regarding the accuracy of partially and fully guided dental implant surgery for the restoration of single-tooth gaps and for partially edentulous and edentulous mandibles [40, 41]. Probst et al. conducted a study to demonstrate the feasibility of 3D T1-weighted bone sequence and 3D T2-weighted short tau inversion recovery (STIR) MRI for computer-assisted template-guided 3D dental implant planning, which was achieved in three-quarters of cases, with the resulting deviation between the virtual planned and the actual implant position being clinically acceptable [13]. Another study supported the feasibility by effectively visualizing all anatomical structures relevant to implant placement comparing various MRI protocols, with 3D HR T1w TSE being superior and showed no significant differences compared to CBCT [31]. Hilgenfeld and colleagues conducted a study in 2020 to demonstrate the usefulness of a multi-slab acquisition with view-angle tilting gradient was used, based on a sampling perfection with application-optimized contrasts using different flip-angle evolution (MSVAT-SPACE) prototype sequence for implant planning and surgical guide fabrication. The MRI datasets were co-registered with the CBCT datasets to evaluate angular discrepancies between planned and surgically guided implant positions. For implant planning, inter-rater and inter-modality agreement for MRI-based treatment planning was excellent, but CBCT-based adjustments to MRI plans were required for implant position in 30% and implant axis in 7%, with almost all guides being suitable for clinical use [42]. Thereby, the mean three-dimensional deviations between MRI- and CBCT-based implant position were 1.1 mm at the entry point and 1.3 mm at the apex, with a mean angular deviation of 2.4°. Furthermore, another study integrating intraoral surface scanning and 3D printing into guided implant surgery has demonstrated the need for an imaging modality that can effectively and accurately visualize mucosal details [43]. However, another evaluation of MSVAT-SPACE-MRI compared to CBCT in assessing the geometric reproducibility of 3D implant planning using a backward planning approach showed higher reproducibility and inter- and intra-rater reliability for CBCT [44]. Obviously, MRI of the oral cavity is challenged by artifacts that may compromise the accuracy and quality of the acquired images due to the presence of dental restorations, depending on their composition and physical properties, field inhomogeneity, breathing, or tongue and deglutition movements [12]. Addressing and minimizing these artifacts is critical to the reliability and interpretability of dental MRI scans in the comprehensive assessment of dental implant therapy. Efforts are therefore being made to optimize MRI protocols, patient preparation, and technical software and hardware. However, in clinical practice, the presence of artifacts in all imaging modalities, including CT and CBCT, needs to be considered as dental restorations causing image artifacts are more prevalent in patients undergoing dental implant surgery due to their tendency to be older, which may affect the accuracy of transferring the surgical plan to the surgical site [45]. In CBCT imaging, metallic artifacts appear as black-and-white streaks, predominantly arising due to X-ray diffraction and photon starvation [46]. In the context of dental implant surgery, the challenge of metallic artifacts extends beyond implant materials themselves. Even the drilling procedures performed during surgery that result in metal debris can cause severe degradation of image quality, as they can become embedded in surrounding tissues [47]. Although artifacts in MRI may originate from similar sources, their underlying physical mechanisms, mainly due to magnetic field distortions, and their visual manifestation vastly differs from X-ray based imaging modalities. While the streak artifacts induced by CBCT are usually less severe in MRI, the presence of these artifacts can still compromise the diagnostic utility of images the images [48]. However, in contrast to titanium implants, zirconia implants showed minimal artifacts and were visualized together with the surrounding tissue with an accurate signal-to-noise ratio [30, 49]. Initial efforts have included the implementation of specific dental MRI protocols, including view angle tilting (VAT) and slice-encoding metal artifact correction (SEMAC) techniques, already successfully established in orthopedics [50] and neurosurgery [51], which have resulted in a reduction in metal artifacts and further improved image quality, but have been associated with a blurring effect [52, 53]. Similarly, in oncological imaging such as PET/MRI in the head and neck region, several studies have aimed to reduce metal artifacts from dental implants by applying deep learning-based assessment to predict the missing information [54]. The results showed promising performance of the proposed approach and reduction of artifacts in the completion of MR images affected by metal artifacts and/or body truncation in PET/MR imaging. Burger et al. developed another algorithm to reduce metal artifacts from dental implants in Dixon-based attenuation map generation using a multi-acquisition variable-resonance image combination sequence, showing robust results in all patients with a 70% reduction in artifact size, allowing MR image-based attenuation correction in critical areas, leading to improved diagnostics [55]. In addition, the use of lower magnetic field strengths in combination with dedicated intraoral or mandibular coils is a promising diagnostic approach that allows fixation of the patient's head and jaw, faster imaging and thus shorter examination times (up to three minutes), which plays a key role in reducing artifacts and making MRI a viable option for clinical applications [16]. However, further research and technological advancements, particularly in light of the ongoing shift toward the use of ultra-low field MRI are still needed to establish standardized approaches aiming at minimizing implant-related artifacts and enhancing the diagnostic potential of dental MRI.

While radiation-free dental MRI offers several advantages and new possibilities in implant dentistry, the results of this systematic review highlight the potential of several MRI protocols for planning, placement, and follow-up of dental implant rehabilitation, with comparable results to CBCT-based surgical planning. The ongoing transition to radiation-free dentomaxillofacial imaging should always consider well-defined case-specific indications and limitations. This approach aims to achieve the best possible patient outcome in a comprehensive, multidisciplinary coordinated, personalized and minimally invasive therapeutic approach. Given the existing heterogeneity in the literature regarding scan parameters and coils used, additional studies, including randomized control trials, are needed to evaluate comparisons between MRI and conventional radiography. Some studies in this systematic review focused on feasibility assessments using MRI alone, highlighting the need for further research. At the same time, adherence to radiation safety guidelines is essential to contribute to an evidence-based understanding of the effectiveness and impact of MR protocols on clinical outcomes. For future meta-analyses, standardization of study designs, outcomes, and methods across studies is essential to ensure a more homogeneous and comparable database. From today's perspective, dental MRI can be used on an indication- and patient-specific basis to replace or complement established X-ray-based imaging modalities such as CBCT or CT. The additional insights derived from soft-tissue information may have a positive impact on surgical planning. This, in turn, might allow better prediction of postoperative outcomes, leading to potentially safer surgical approach and minimizing postoperative discomfort or complications. However, with increased accessibility, improved cost-effectiveness and considering the improved benefit-risk ratio, the use of UTE or ZTE protocols with clinically acceptable acquisition times represents a promising alternative that can be used for oral rehabilitation planning, enabling advanced treatment options such as guided implantation.

## Conclusions

In conclusion, this systematic review investigates the existing literature on the feasibility and accuracy of MRI in dental implantology. The analysis focuses on the impact of MR protocols and technical features and provides insight into their indications and limitations for perioperative imaging. The results emphasize the significant contribution of dental MRI in the assessment of critical clinical parameters, including osseous tissue structure, bone dimensions, and proximity to vital structures. While conventional X-ray-based imaging techniques remain the gold standard, Black Bone MRI and MSVAT-SPACE MRI protocols, which offer improved hard and soft tissue resolution and higher sensitivity in detecting pathologic changes compared to conventional X-ray-based modalities, may establish themselves as a valuable alternative in targeted cases where CBCT is insufficient. The results of this review indicate that further studies, including randomized control trials, are needed to evaluate the efficacy and impact of MRI protocols on clinical outcomes. Therefore, standardization of study designs is essential for future meta-analyses to ensure a homogeneous and comparable database. While the benefits of MRI in implant dentistry are currently being demonstrated, further research is needed to evaluate its long-term efficacy, cost-effectiveness, and broader applicability. However, implementing dental MRI into the perioperative workflow has the potential to redefine treatment strategies, increase precision, and improve patient outcomes while minimizing radiation exposure.

## Data Availability

The datasets generated and analyzed during the current study are available from the corresponding author on reasonable request.
